# Molecular Population Genetics and Evolution of the Chagas’ Disease Vector *Triatoma infestans* (Hemiptera: Reduviidae)

**DOI:** 10.2174/13892029113149990006

**Published:** 2013-08

**Authors:** Beatriz A. García, Alicia R. Pérez de Rosas, María J. Blariza, Carla G. Grosso, Cintia J. Fernández, María M. Stroppa

**Affiliations:** Cátedra de Bioquímica y Biología Molecular, Instituto de Investigaciones en Ciencias de la Salud (INICSA, UNC-CONICET), Facultad de Ciencias Médicas, CONICET and Universidad Nacional de Córdoba, Argentina

**Keywords:** Chagas' disease vector, DNA sequences, Genetic structure, Microsatellites, Phylogeography, Triatoma infestans

## Abstract

Triatoma infestans (Klug) is the main vector of Chagas’ disease in the Southern Cone of Latin America between the latitudes 10° S and 46° S. The long-term effectiveness of the control campaigns is greatly dependent upon the vector population structure. Mitochondrial DNA (mtDNA) genes have been used in a number of T. infestans population genetic analyses. However, the maternally inherited markers as well as nuclear ribosomal DNA analyzed until the present exhibited low or limited levels of variation. Analyses based on microsatellite markers strongly supported the existence of some type of stratification in T. infestans populations and supported the hypothesis of vector population recovery from survivors of the insecticide-treated areas, highlighting the value of population genetic analyses in assessing the effectiveness of Chagas’ disease vector control programmes. Although phylogeographic studies have generally suggested a Bolivian Andean origin of T. infestans, they recovered two reciprocal monophyletic groups of T. infestans and Bolivian populations who were not basal as expected for an ancestral group. In addition, a non-Andean origin could not be excluded by mtDNA genealogies that included sylvatic bugs from Gran Chaco. On the other side, mitochondrial and microsatellite markers supported the hypothesis of two independent migration events of colonization and secondary contacts in southern South America. Since the phylogenetic analyses remain inconclusive, more sequences, not only from mitochondrial genes but also from nuclear genes, need to be examined.

## INTRODUCTION

Chagas' disease (American trypanosomiasis) is a public health problem with high socio-economic impact in Latin America, where about 7 to 8 million people are affected by the malady [1]. The disease is caused by infection with *Trypanosoma cruzi* (Chagas), a protozoan transmitted by hematophagous insects of the subfamily Triatominae (Hemiptera: Reduviidae). Transmission requires the contact of host skin or mucosa with the vector´s ejecta containing metacyclic forms of *T. cruzi*. The adults of both sexes and the five nymphal instars of the species belonging to Triatominae are bloodsuckers and at least one full blood meal is necessary for each molt. Therefore, the insects are potential vectors of *T. cruzi* from their earliest stages since they may acquire the infection from their first meal. At present, 147 species of Triatominae are formally recognized [2]. The vectors belonging to this subfamily are extensively distributed from the United States to Argentina approximately between latitudes 46˚N and 46˚S. From an epidemiological standpoint, the most important species of triatomines involved in the transmission of Chagas’ disease are those that combine a high degree of adaptation to the domestic environments, have a wide geographical distribution, possess a high vectorial capacity, and are anthropophilic [3]. A general evolutionary trend for members of the subfamily Triatominae, from exclusively sylvatic species to those well adapted to live in human habitats, has been suggested. Considering their different habitats, only a few species, having a high degree of adaptation to the domestic environment, are recognized as effective vectors of trypanosomiasis in humans. Among them, *Triatoma infestans* (Klug) is one of the species most closely associated with man, widely distributed, and consequently of public health importance. It is the main transmitter of Chagas' disease in the Southern Cone of Latin America between the latitudes 10° S and 46° S. Across its distribution *T. infestans* is primarily restricted to domestic and peridomestic environments such as human dwellings, chicken coops and pig or goat corrals.

Currently, neither satisfactory treatment of the malady nor a preventive vaccine against *T. cruzi* is available. The measures taken to reach the interruption of transmission of this parasitic and infectious disease rely on the vector control by insecticide treatment of infested dwellings; however, though important progress has been made by the Southern Cone Initiative, the long-term support for the surveillance phase is a new challenge for the region. The important accomplishments reached by the vector control programmes have been the elimination of *T. infestans* in Uruguay and Chile [4] and the interruption of the *T. cruzi* transmission by *T. infestans* in Brazil. However, high levels of *T. infestans* reinfestation were observed in Argentina, Bolivia, and Paraguay after spraying [5]. In Argentina, the National Chagas Control Program established in 1962 pursued the elimination of domestic and peridomestic populations of *T. infestans* by insecticide spraying. From 1962 to 2000, almost all the localities within the endemic area received insecticide treatment, but in most of them the entomological surveillance was not maintained after 20 years of the implemented programme [6]. As a consequence, resurgence of vector transmission of the disease was observed in the endemic region, where new acute cases have been detected since the year 2000 [7]. Vector control has proven to be difficult because of the variability and extension of endemic areas and the long period of control necessary to prevent the recovery of treated bug populations. In this respect, it was reported that domiciliary re-infestation returned to pre-spraying levels in three or four years in a locality of Santiago del Estero province (Argentina) where additional spraying of insecticide was not applied [8]. The long-term effectiveness of the control campaigns is greatly dependent upon the vector population structure. The macro- and microgeographical genetic analyses of populations of *T. infestans* provide new basis for understanding the evolutionary history, migration patterns, genetic structure and dynamics of vector populations and resolving questions on processes such as dispersal and recolonization of the species that directly affect the efficiency of control efforts. Particularly, the genetic analysis of vector populations may be useful in entomological surveillance of Chagas vectors control programmes, since it may give information about the source of the insects in reinfested areas. On the other side, it has been detected in some places that vector control failure is caused by resistance to pyrethroid insecticides [9-13]. In this regard, the knowledge of the structure of vector populations is also important for the management of the resistance. Previous population analyses based on allozymes of *T. infestans* produced contradictory results. Dujardin *et al.* [14-16] and Pereira *et al.* [17] reported low polymorphism inferred from results on two or three polymorphic loci, which allowed weak genetic population inferences. Contrary to very low degree of polymorphism, García *et al.* [18, 19] detected an important level of genetic variability in laboratory colonies, which could be explained by differences in methodology [18]. Among the newer genetic tools, mainly DNA techniques, have been used to address fundamental questions for effective vector control providing valuable information of central importance for the development of control strategies.

## MACROGEOGRAPHIC AND MICROGEOGRAPHIC GENETIC STRUCTURE

Mitochondrial DNA (mtDNA) genes have been used in a number of *T. infestans* population genetic analyses. Analyses of mitochondrial cytochrome B gene sequences revealed four haplotypes in nine geographical populations of *T. infestans* from Argentina, Bolivia and Brazil, and did not show variations within populations [20]. Sequence comparisons of mtDNA fragments of the 12S and 16S ribosomal RNA genes revealed 13 haplotypes among 40 *T. infestans* from five natural populations of Argentina, 10 of which presented variable nucleotide sites in the 16S sequence fragment and eight showed variable nucleotide sites only in that fragment [21]. A total of 10 private haplotypes were found in four of the populations analyzed, suggesting low current levels of genetic exchange. The haplotypic diversity and the private haplotypes found in two of the localities studied suggested that the recovery of insecticide-treated populations came from survivors of the same area. In both, the intraspecific work cited above as well as in interspecific studies [22-26], the 16S sequences were variable and consequently informative. Subsequently, variation in the mtDNA 16S ribosomal RNA gene in populations of *T. infestans* was surveyed. DNA sequence comparisons yielded 18 haplotypes among 130 individuals from 16 localities that represented a large portion of the range of *T. infestans* in Argentina [27]. A total of 15 haplotypes were present exclusively in one of the populations. These private haplotypes, found in the majority of the populations, also suggested limited current levels of gene flow. Analysis of mtDNA 16S sequences uncovered substantial genetic variation among* T. infestans *populations; haplotype and nucleotide diversities varied from 0 to 0.84 and 0 to 0.29%, respectively. Genetic differentiation between populations was not significant in most of the comparisons with a few exceptions. However, these results were based on a genetic marker that presented low or no variation in the populations analyzed and should not be taken as conclusive evidence that *T. infestans* populations are genetically undifferentiated.

On the other hand, Piccinali *et al.* [28] analyzed a fragment of the mitochondrial gene cytochrome oxidase (COI) and detected a total of 37 haplotypes among 244 insects; 32 haplotypes among 207 bugs from Argentina, five among 25 from Bolivia, one among seven from Perú, and two among five bugs from Uruguay. In Argentina, the haplotype diversity ranged between 0.23 and 0.84 and nucleotide diversity between 0.05 and 0.67%. Pairwise comparison between Argentinian provinces indicated the existence of a strong population structure. However, in this study samples from different localities belonging to one province were pooled for the analysis and consequently the effect of considering together different populations could not be ruled out in the interpretation of these results.

The maternally inherited markers analyzed exhibited low or limited levels of variation [20, 21, 27, 28]. Similarly, another study based on 34 specimens of *T. infestans* from different localities of seven countries (Peru, Bolivia, Brazil, Chile, Uruguay, Paraguay, and Argentina), using nuclear ribosomal DNA (ITS-1 and ITS-2) sequences, revealed very little genetic variation [29]. Among the nuclear markers, microsatellites have permitted greater resolution of genetic variation of populations. These markers are potentially useful because they are abundant, highly polymorphic, and provide information about the state of specific loci, facilitating a number of population-genetic inferences [30, 31].

García *et al.* [32] isolated 93 microsatellite loci from partial genomic libraries of *T. infestans;*, 30 of these loci were amplified and 10 for which different allele types could be resolved clearly were selected for genotyping. The high degree of intra-population variation detected in the microsatellite loci analyzed suggested that these markers provide a valuable molecular tool for genetic analysis of *T. infestans* populations [32]. These variable nuclear markers were firstly used to study 598 insects from 19 populations of *T. infestans* from Argentina [33]. Departures from Hardy-Weinberg expectations due to an excess of homozygotes suggested population subdivision. Subdivision of the population into groups differing in allele frequencies produces an effect similar to that originated by inbreeding, even if random mating is the rule within each subpopulation (Wahlund effect) [34]. Two microgeographic analyses were performed to verify if *T. infestans* populations are subdivided. One of them was carried out comparing samples of *T. infestans* obtained within four houses of one of the localities analyzed (Siete Árboles, Chaco province), where there were no peridomestic environments such as chicken coops and pig or goat corrals, usually invaded by *T. infestans* [33]. The heterogeneous distribution of allele frequencies suggested the existence of some type of stratification in the population, e.g. subdivision into breeding units with restricted possibilities of genic exchange that may explain the observed heterozygote deficiency. The existence of subdivision in *T. infestans* populations was also strongly confirmed using the same markers with specimens collected from three peridomiciliary sites in Medanitos (Catamarca province), where specimens within human dwelling were not detected [35]. In agreement with the results that support the existence of stratification in *T. infestans* populations, three and two different genetic clusters were identified in Siete Arboles (Chaco province) and Medanitos (Catamarca province), respectively. One should expect mobile species not to show genetic differentiation over short distances as indicated by the results of the microgeographical analysis, unless geographical features and/or very strong selection pressure prevent gene flow. In this regard, *T. infestans* has been characterized as an insect with restricted dispersal over short distances and usually remains in the same house or in its immediate vicinity during its lifetime. Besides, a significant excess of homozygotes was observed in the samples from two of the four houses analyzed in Siete Árboles and two of the three houses analyzed in Medanitos, suggesting inbreeding and/or a high degree of subdivision in the population [35].

Simultaneously, nine out of the 10 microsatellite loci previously isolated and characterized [32] were also used by Richer *et al.* [36] to assess the dispersal ability of wild Andean *T. infestans* at microgeographical scale and the possibility for wild populations to actively recolonize insecticide treated villages. At base to the levels of genetic differentiation detected between populations of *T. infestans* in the Bolivian Andes, the authors inferred restricted genetic interchange between close but distinct sylvatic sites, which supported the hypothesis that wild *T. infestans* does not disperse by flying at high altitude. They also inferred restricted gene flow between domestic and sylvatic populations, which suggested limited short term role of wild insects in the process of recolonization of insecticide treated houses in the Andes. Pizarro *et al.* [37] also used the microsatellite characterized by García *et al.* [32] to analyze the genetic structure of *T. infestans* populations from an ecologically diverse but geographically small valley-mountain environment in the department of Chuquisaca in Southern Bolivia. There they found significant genetic differentiation at different hierarchical geographic level; between low altitude East and high altitude West, among the five communities analyzed, and among houses from one of them, where they observed significant differentiation between domestic and peridomestic populations. Five genetic clusters within the five communities were identified. One of them was a mixture of insects from three close localities and the other four clusters contained insects from primarily one locality. In agreement with the results obtained in other vector population genetic studies [33, 35, 36], the significant population structure detected supports the hypothesis of reduced migration of *T. infestans*. Besides, the high degree of genetic structure at small geographic scale inferred by cluster analysis and assignment tests together to demographic data suggested that the domiciliary re-infestation is from residual population and from peri-domicile structures.

On the other side, Pérez de Rosas *et al.* [33] evaluated the possible effect of eradication campaigns on the genetic structure of the vector populations, comparing levels of genetic variability in natural populations of *T. infestans* from areas with different elapsed periods since the last insecticide treatment and from areas that never received treatment. *T. infestans* populations from insecticide-treated localities seemed to have retained a substantial proportion of genetic diversity. The levels of genetic diversity observed in the majority of the populations from insecticide treated areas were surprising considering that insecticide treatments produce severe population reductions (bottlenecks) and that random genetic drift should cause loss in genetic variation. The authors considered the possibility that the population crash during the bottleneck would have been overestimated. Allelic diversity decreases faster than heterozygosity, when a population experiences a reduction of its effective size, and generally develops a heterozygosity excess at selectively neutral loci [38]. The results obtained by heterozygosity tests did not support the occurrence of recent bottleneck events. However, the heterozygosity tests used to detect recent bottleneck events rely on the assumption that each sample is representative of a population with no immigration and no population substructure. If the population is subdivided into several reproductive units, this method may not be able to show evidence of bottlenecks. In this respect, the studies mentioned above [33, 35-37] supported the existence of subdivision in *T. infestans* populations. It was suggested that, since the populations of *T. infestans* are subdivided, a population bottleneck would result in several independent genetic drift effects that could randomly preserve different combinations of alleles in each subpopulation; if independent genetic drift events in the population were followed by a rapid population growth, high levels of genetic diversity could be preserved. The results obtained by Pérez de Rosas *et al.* [33] supported the hypothesis of vector population recovery from survivors of the insecticide-treated areas, highlighting the value of population genetic analyses in assessing the effectiveness of Chagas’ disease vector control programmes.

Spatial genetic structure was also detectable at macrogeographical level [33, 39]. This result indicated significant deviation from a pattern of unrestricted gene flow, suggesting that the magnitude of gene flow is not sufficiently large to mask differences eventually produced by genetic drift. This is not surprising since the limited dispersal described for *T. infestans* would tend to accentuate the genetic differentiation through the process of genetic drift. Moreover, significant isolation by distance, with nearby sites apparently exchanging more genes than distant ones, was detected. However, some sites exhibited deviation from a pattern of isolation by distance, suggesting that allele frequencies in each of these sites drift independently of the geographic distances separating them, probably factors other than geographical distance could be involved in generating the structure of populations. The majority of *T. infestans* populations studied belonged to areas with different elapsed periods since the last insecticide treatment and only three were from areas that never received treatment. It was considered that local differentiation by genetic drift during the reduction of population size by insecticide treatment could create significant structuring independent of distance. Pérez de Rosas *et al.* [33] suggested that the higher degree of divergence detected between some geographically close localities was due to the short time elapsed from the last insecticide intervention in at least one of the locations involved in the pair compared. The authors indicated that probably there has not been enough time for gene flow to prevent the level of differentiation detected between these populations that could have diverged because of genetic drift from isolation during population bottlenecks. Therefore, as a consequence of insecticide treatment, genetic drift may have played an important role in genetic differentiation among populations.

In order to study the genetic structure of *T. infestans* populations from a region with the same insecticide treatment, Pérez de Rosas *et al.* [35] examined the genetic structure of *T. infestans* populations using the same 10 microsatellite markers previously isolated [32] from six localities belonging to Catamarca province (Argentina), all of which received the last insecticide treatment five years before the sample collection. The results confirmed that populations of *T. infestans* are highly structured. Spatial genetic structure was detectable at macrogeographical and microgeographical levels from this region with the same insecticide treatment. Significant isolation by distance was also detected and seven different genetic clusters were identified. The six populations analyzed corresponded to six distinct clusters; one of these populations showed higher heterogeneity with approximately 30% of individuals corresponding to the seventh cluster identified. In this study, comparisons of the levels of genetic variability between two temporal samples from one of the localities analyzed were also carried out to assess the impact of the insecticide treatment. A second sample from Medanitos, collected 16 months after the capture of the first sample and subsequent to insecticide treatment, was also analyzed. The genetic diversity of the population was not significantly affected after insecticide use since different genetic parameters (allele number, observed and expected heterozygosities) remained stable; however, loss of low frequency alleles and no previously found alleles were detected. The authors stated that one explanation for the absence of diversity loss after insecticide treatment may be the size of the remnant population. The effective population size (*N_e_*) estimated for the population 16 months after insecticide treatment was substantially lower than the *N_e_* estimated before that treatment. Nevertheless, the population had only been studied for two generations after insecticide treatment and the remnant population size probably stayed high enough to maintain its genetic diversity at the pre-treatment level.

On the other side, Marcet *et al.* [40] identified and characterized 13 microsatellite loci of *T. infestans*, of which nine were considered suitable for population genetics studies. These loci plus a 10th locus isolated later were used to analyze 352 insects collected in 21 houses of 11 rural communities from two neighboring rural areas in Santiago del Estero province (Argentina) that differed in their history of vector control [41]. This study suggested that the long-term control interventions affected the population genetic structure of the vector. *T. infestans* populations from the core area, which were subjected to recurrent vector control actions, were highly structured and presented eight genetic clusters. Contrary to this, bug populations from peripheral area with sporadic vector control appeared to be less structured and less differentiated than those from the core area. Concordantly with the work mentioned above [35], the authors indicated that substructure within houses accounts for the significant heterozygote deficit observed in some houses. Besides, the results indicated that houses received immigrants from more than one source and were consistent with active insect dispersal within and among communities. In a new study, carried out in the same geographical area of the Argentinean Chaco, were detected six low-density sylvatic foci with 24 specimens of *T. infestans*. Mitochondrial cytochrome B and/or COI gene sequences confirmed the identification of 20 of these sylvatic bugs as *T. infestans*. These mtDNA fragments and the microsatellite markers indicated the occurrence of unrestricted gene flow between sylvatic and domestic or peridomestic *T. infestan*s populations from that area [42]. The authors concluded that sylvatic habitats may provide transient or permanent refuge after insecticide treatment and therefore may be the source for domestic or peridomestic reinfestation. It was also suggested that the occurrence of sylvatic foci of *T. infestans* in the Gran Chaco may be an additional problem for vector control.

Recently, Pérez de Rosas *et al.* [43] proposed a multilocus approach to examine fine scale patterns of genetic structure and dispersal in *T. infestans*. With this purpose, a total of 314 insects from 22 domestic and peridomestic sites from one locality of Catamarca province (Argentina) were typed for 10 polymorphic microsatellite loci previously isolated [32]. Significant levels of genetic differentiation were observed among all collection sites, including the different sampled sites within the same house. The scale of structuring detected in the spatial autocorrelation analyses showed that dispersal typically occurs on the scale of approximately 400 m. These results suggest that in order to reach a higher vector control effectiveness, insecticide treatment and surveillance should be extended within a radius of 400 m around the infested area, which would prevent the reinfestation process after insecticide spraying.

## PHYLOGEOGRAPHICAL INFERENCES

Based on arqueological findings and historical reconstructions, it has been suggested that the origin of *T. infestans* was from Andean highland in Bolivia, where after it was introduced into human dwellings (~ 4500 years ago) the species was dispersed from this center throughout Latin American. As part of its dispersion associated with human migration it is estimated that, the vector would have invaded southern Brazil arriving from Argentina and Paraguay, and from this region it would have dispersed to the northeast of Brazil. According to this hypothesis, its current distribution may be interpreted as the result of the dispersal of the species from the site of origin in the Cochabamba valley of Bolivia, coupled with recent and rapid changes in the spread of *T. infestans* related to the activities of man, particularly between the 19th and 20th centuries [44].

Mitochondrial DNA (mtDNA) genes have been recognized particularly useful for phylogeographic studies in many species of insects, which involve migration pattern inferences [45]. Initially, four haplotypes determined by eight variable nucleotide sites from a fragment of the mitochondrial cytochrome B gene were detected in *T. infestans* populations [20]. Two clusters could be identified from these data; one involved samples from Argentina and Brazil and the other included samples from Bolivia. The phylogenetic analysis suggested the separation of the hypothetical ancestral form of the populations from Bolivia from the hypothetical ancestral form of the populations from Argentina and Brazil. Besides, Monteiro *et al.* [20] were in agreement with the hypothesis that supports the invasion of Brazil from insects of Argentina.

Subsequently, Panzera *et al.* [46] identified two chromosomic allopatric groups in *T. infestans* populations, named Andean and non-Andean. These authors detected differences in heterochromatin that have been considered the main cause of the variation in the DNA content between both groups, with approximately 30 % more heterochromatin in the Andean insects. On the basis of the observation that Andean samples came from geographic regions generally above 1800 m, whereas non-Andean populations were mainly from localities below 500 m, these authors suggested that heterochromatin and DNA variation reflect adaptative genomic changes that contribute to the ability of *T. infestans* to survive and reproduce in environments with different altitudes; populations with large genome would be better adapted to highlands (Andean regions), whereas populations with smaller genomes would do better in lowlands (non-Andean regions). 

Phylogenetic inferences carried out, using nuclear rDNA (ITS-1 and ITS-2) sequences, supported the previously suggested origin of *T. infestans* in Bolivia highlands [44] and two different dispersal lines, one throughout Andean regions of Bolivia and Perú, and the second in non-Andean lowlands of Chile, Paraguay, Argentina, Uruguay and Brazil [29]. In this study, composite ITS-1-ITS-2 haplotypes indicated a differentiation between western (Andean) and eastern (non-Andean) populations of *T. infestans*. However, Quisberth *et al.* [47] observed ITS-2 and mitochondrial cytochrome B haplotypes in the Gran Chaco (Bolivia) previously found only in Andean areas. The authors concluded that the presence of these haplotypes in domestic populations from four villages of the Gran Chaco would be the result of the human passive transport of insects from the Andes to the Gran Chaco. On the other side, Piccinalli *et al.* [28] found a higher degree of variation in a fragment of the mitochondrial COI gene than in previous mtDNA based studies [20, 21, 27]. A maximum parsimony analysis of the haplotypes showed an Argentinean haplotype as the most basal lineage and the presence of an Argentinean/Uruguayan and a Bolivian/Peruvian clade. The last was further subdivided into two clusters; one including the sylvatic dark morph haplotype from the Bolivian Chaco and the other the domestic and sylvatic Andean haplotypes. The haplotypes from Uruguay were also present in Argentina, and within this last country the tree was not well resolved. In concordance with the mitochondrial and nuclear genes analyzed previously [20, 29], the phylogeny of COI haplotypes supported the hypothesis of an Andean and a non-Andean *T. infestans* allopatric group.

Although phylogeographic studies have generally suggested a Bolivian Andean origin of *T. infestans*, they recovered two reciprocal monophyletic groups of *T. infestans* [20, 28, 29], and Bolivian populations were not basal as expected for an ancestral group. Moreover, the recent discovery of sylvatic *T. infestans* not only in the Bolivian Andean valleys challenges this hypothesis. This finding prompted the hypothesis that the most ancient populations would be those of the dry subtropical Chaco forest in southeastern Bolivia, Paraguay and the north of Argentina [48]. To this respect, analysis of mitochondrial cytochrome B haplotype genealogies in Bolivian sylvatic populations showed that the non-Andean (Gran Chaco) haplotypes would have derived from the Andean ones, supporting an Andean origin of *T. infestans*. However, a non-Andean origin could not be excluded; first because only a single population in Gran Chaco was analyzed and second because higher genetic variability was detected in this population than in all the Bolivian Andean valley populations studied. Since in older populations more time would allow the accumulation of a higher level of genetic variation, the non-Andean region could be the center of origin and dispersal [49]. In concordance, analyses carried out by Piccinali *et al.* [50] using mitochondrial COI sequences, were consistent with the presence of ancestral haplotypes in sylvatic bugs from the Argentinean Chaco. In this study, the occurrence of sylvatic basal haplotypes in phylogenetic analyses that included Bolivian haplotypes and the higher level of genetic variability detected in sylvatic bugs than in domestic and peridomestic *T. infestans* populations from Argentina [28], supported the hypothesis of the Chaco region as the area of origin of *T. infestans*. 

On the other side, the maternally inherited markers analyzed until the present in *T. infestans*, either exhibited limited levels of variation or have not been very useful for phylogeographic inferences of the Chagas’ disease vector in Argentina [20, 21, 27, 28, 51]. Genetic variation in *T. infestans* observed at the 16S locus [27] as well as at cytochrome B and COI mtDNA sequences [20, 28, 51] was considerably less than the one revealed by analysis of microsatellite markers [33, 35-37, 39, 41]. The discrepancy in magnitude of variation of both mtDNA sequences and microsatellites may be related to differences in rate and pattern of mutations of these markers. One explanation for the limited variation observed in the mtDNA genetic markers is a low mutation rate.

A multilocus approach with 10 microsatellite loci was performed to infer the phylogeography and migration patterns from different *T. infestans* populations covering almost the entire species range in Argentina [39]. Microsatellite data set of 836 individuals from 27 populations of *T. infestans* was analyzed. Pérez de Rosas *et al.* [39] identified seven different genetic clusters; six out of the seven were distinct, almost homogeneous clusters distributed across specific geographic locations. While 11 populations corresponded to the six different, nearly homogeneous clusters (more than 80% of ancestry shared among individuals), 16 populations resulted more heterogeneous presenting mix of different clusters. Most of the first 11 populations formed three almost homogeneous clusters detected in the west of Argentina. These three groups of populations practically did not share ancestry among them, suggesting a lower level of gene flow and consequently a higher population differentiation by drift in this region. Besides, these populations belonging to localities geographically close to the Andean highlands almost did not share ancestry with the rest of the populations analyzed. Considering the findings of Panzera *et al.* [46] and Bargues *et al.* [29], Pérez de Rosas *et al.* [39] suggested that the populations from the western area of Argentina, most of which are located between 1000 and 1700 m, could have been established from the dispersal line of *T. infestans* that would have arrived to Argentina through the Andes. The authors also suggested that the presence in this area of populations that share some degree of ancestry with eastern populations would imply process of secondary contact between populations from the different dispersal lines. On the other side, it was considered that probably most of the other populations analyzed may have derived from the dispersal line of *T. infestans* in non-Andean lowlands associated to human migratory movements. These populations, which presented lower degree of genetic differentiation, showed higher heterogeneity (mix of different clusters) sharing different percentages of ancestry. It was suggested that the human internal migratory movement linked to regional economies, presumably associated with passive dispersal, would allow to maintain a higher genetic exchange between these populations of *T. infestans*.

In summary, the last study favored the hypothesis of two independent migration events of colonization in Argentina and secondary contacts. Based on the analysis of a fragment of the mitochondrial COI gene, Torres-Pérez *et al.* [52] assessed alternative biographic scenarios of dispersal of *T. infestans* using coalescence simulations and phylogeographic structure of this species in Chile. They also supported the hypothesis of two independent migration events of *T. infestans* in southern South America including a dual-origin of *T. infestans* in Chile.

## CONCLUSIONS

Modern molecular approaches, employing DNA technology, afford vast new possibilities for studies of genetic population structure and evolution of *T. infestans*. The origin of reinfestations after insecticide treatment has remained controversial. The research supports the hypothesis of vector population recovery from survivors of the insecticide-treated areas, highlighting the value of population genetic analyses in assessing the effectiveness of Chagas’ disease vector control programmes. Moreover, the inferences made from these analyses in *T. infestans* populations demonstrated to be important, providing a complementary approach to help improve vector control strategies. On the other side, it is necessary to take into account the possible effect of the eradication campaign on the genetic structure of the vector populations since insecticide treatment would tend to accentuate the genetic differentiation among populations through the process of genetic drift. Further analyses should be undertaken to clarify if this effect may have epidemiological implications. The amount of genetic variation and differentiation through a species range is influenced by several factors, current and historical, which are often difficult to disentangle. It is possible that historical events, such as population bottlenecks, local extinctions and founder event, have probably played a major role in shaping their population genetic structures. Further analyses should be undertaken, since it is of considerable public health interest to know for example if genetic differentiation may be or could become associated with differences in the capacity of populations to act as vectors. More sequences, not only from mitochondrial genes but also from nuclear genes, need to be examined. In this respect, Fernández *et al.* [53] analyzed recently the variation in mitochondrial NADH dehydrogenase subunit 5 (ND5) and NADH dehydrogenase subunit 4 (ND4) genes in *T. infestans*. Based on their results it was inferred that the amplified regions should be useful for genetic analysis of *T. infestans* populations.
